# Association between Higher Circulating Leucine-Rich α-2 Glycoprotein 1 Concentrations and Specific Plasma Ceramides in Postmenopausal Women with Type 2 Diabetes

**DOI:** 10.3390/biom12070943

**Published:** 2022-07-05

**Authors:** Alessandro Mantovani, Alessandro Csermely, Elena Sani, Giorgia Beatrice, Graziana Petracca, Gianluigi Lunardi, Stefano Bonapace, Giuseppe Lippi, Giovanni Targher

**Affiliations:** 1 Section of Endocrinology, Diabetes and Metabolism, Department of Medicine, University and Azienda Ospedaliera Universitaria Integrata of Verona, 37126 Verona, Italy; alessandro.mantovani@univr.it (A.M.); csermelyale@gmail.com (A.C.); elenasani@live.it (E.S.); giorgiabeatricejb@gmail.com (G.B.); grazianapetracca1@gmail.com (G.P.); 2 Clinical Analysis Laboratory and Transfusion Medicine & Clinical Pharmacology, “IRCCS Sacro Cuore-Don Calabria” Hospital, 37024 Negrar, Italy; gianluigi.lunardi@sacrocuore.it; 3 Division of Cardiology, “IRCCS Sacro Cuore-Don Calabria” Hospital, 37024 Negrar, Italy; stefano.bonapace@sacrocuore.it; 4 Section of Clinical Biochemistry, Department of Medicine, University and Azienda Ospedaliera Universitaria Integrata of Verona, 37126 Verona, Italy; giuseppe.lippi@univr.it

**Keywords:** ceramides, cytokines, diabetes mellitus, inflammation, leucine-rich α-2 glycoprotein 1, LRG1

## Abstract

Background: Although ceramides are involved in the pathophysiology of cardiovascular disease and other inflammation-associated disorders, there is a paucity of data on the association between plasma ceramides and inflammatory biomarkers in type 2 diabetes mellitus (T2DM). Therefore, we explored whether there was an association between plasma leucine-rich α-2 glycoprotein 1 (LRG1) concentrations (i.e., a novel proinflammatory signaling molecule) and specific plasma ceramides in postmenopausal women with T2DM. Methods: We measured six previously identified plasma ceramides, which have been associated with increased cardiovascular risk [plasma Cer(d18:1/16:0), Cer(d18:1/18:0), Cer(d18:1/20:0), Cer(d18:1/22:0), Cer(d18:1/24:0) and Cer(d18:1/24:1)], amongst 99 Caucasian postmenopausal women with non-insulin-treated T2DM (mean age 72 ± 8 years, mean hemoglobin A1c 6.9 ± 0.7%), who consecutively attended our diabetes outpatient service during a 3-month period. Plasma ceramide and LRG1 concentrations were measured with a targeted liquid chromatography-tandem mass spectrometry assay and a Milliplex^®^ MAP human cardiovascular disease magnetic bead kit, respectively. Results: In linear regression analyses, higher plasma LRG1 levels (1st tertile vs. 2nd and 3rd tertiles combined) were associated with higher levels of plasma Cer(d18:1/16:0) (standardized β coefficient: 0.289, *p* = 0.004), Cer(d18:1/18:0) (standardized β coefficient: 0.307, *p* = 0.002), Cer(d18:1/20:0) (standardized β coefficient: 0.261, *p* = 0.009) or Cer(d18:1/24:1) (standardized β coefficient: 0.343, *p* < 0.001). These associations remained significant even after adjusting for age, body mass index, systolic blood pressure, total cholesterol level, hemoglobin A1c, insulin resistance and statin use. Conclusions: The results of our pilot exploratory study suggest that higher plasma LRG1 concentration was associated with higher levels of specific high-risk plasma ceramide molecules in elderly postmenopausal women with metabolically well-controlled T2DM, even after adjusting for known cardiovascular risk factors and other potential confounding variables.

## 1. Introduction

In the past decade, it has become increasingly clear that cardiometabolic disorders such as obesity, type 2 diabetes mellitus (T2DM) and cardiovascular disease (CVD) can occur when the inflammatory response is dysregulated and dysfunctional [1,2]. Leucine-rich α-2 glycoprotein 1 (LRG1) is a novel multifunctional proinflammatory signaling molecule, belonging to a highly conserved member of the family of leucine-rich repeat (LRR) proteins, which is mainly involved in protein–protein interaction, signaling and cell adhesion [3,4,5]. LRG1 has been initially described as an important player in pathological angiogenesis [3,4,6]. Experimental evidence has shown that LRG1 may also play a role in the pathophysiology of several chronic diseases [3], such as T2DM [7], obesity [5], CVD [8] and other inflammation-associated conditions [9,10].

There is now accumulating evidence showing that the sphingolipid pathway is a key player in the chronic inflammatory response and associated diseases [2,11]. Although sphingolipids mainly act as structural lipids in cellular membranes, they may also play a role as second messengers in many intra-cellular and inter-cellular signaling pathways [2,11]. In this regard, ceramides are the main secondary messengers of the sphingolipid pathway and can modulate a variety of metabolic and proinflammatory pathways that are potentially involved in the pathophysiology of CVD and other inflammation-associated disorders [2,11]. For example, a meta-analysis of seven longitudinal studies (involving a total of ~30,000 participants) reported that specific plasma ceramides [especially higher levels of plasma Cer(d18:1/16:0), Cer(d18:1/18:0) and Cer(d18:1/24:1)] were strong predictors of adverse cardiovascular outcomes both in the general population and in patients with established or suspected ischemic heart disease (IHD) [12].

Presently, there is a paucity of published data regarding the potential association between the levels of previously identified high-risk plasma ceramide molecules and proinflammatory biomarkers in individuals with T2DM or those with suspected IHD [2,13,14,15]. In particular, no information is available to date on the association between specific high-risk plasma ceramide molecules and circulating levels of LRG1. We believe that this topic could be of clinical relevance and should be deeply investigated, as it could provide new insights into the putative biological mechanisms underlying the associations between distinct plasma ceramides and CVD or other inflammation-associated conditions.

Therefore, the main aim of our pilot exploratory study was to examine whether specific plasma ceramide species, which have been previously associated with increased CVD risk, were significantly associated with higher plasma LRG1 concentrations in postmenopausal women with T2DM, i.e., a group of individuals at high risk of developing adverse cardiovascular events and other inflammation-associated conditions.

## 2. Materials and Methods

### 2.1. Patients

We consecutively enrolled 99 Caucasian postmenopausal women with established T2DM, who regularly attended our diabetes outpatient service during a 3-month period (from October to December 2017). We excluded (a) patients with a prior history of cirrhosis or chronic liver diseases, cancer and end-stage renal disease; (b) patients with significant alcohol consumption (>20 g/day); (c) those treated with insulin, hormone replacement therapy or anti-osteoporotic drugs; and (d) those with acute infections. We adopted these exclusion criteria because this study was initially designed for examining the association between nonalcoholic fatty liver disease, bone turnover biomarkers and bone mineral density in postmenopausal women with non-insulin-treated T2DM [16].

### 2.2. Clinical and Laboratory Data

Body mass index (BMI) was measured as kilograms divided by the square of height in meters. Blood pressure was measured with a standard sphygmomanometer after the subject had been seated quietly for at least 5 min. We also calculated the pulse pressure using the following equation: pulse pressure = (systolic blood pressure—diastolic blood pressure). Subjects were considered to have arterial hypertension if their blood pressure was ≥140/90 mmHg or if they were treated with any anti-hypertensive agents. Information on smoking history and use of medications was obtained from all patients by interviews during medical examinations. Venous blood samples were drawn in the morning after an overnight fast. Serum creatinine (measured with a Jaffé rate blanked and compensated assay), glucose, lipids, alanine aminotransferase (ALT) and other biochemical blood parameters were assayed using standard laboratory procedures (Siemens Dimension Vista, Siemens Diagnostics, Erlangen, Germany) at the central laboratory of our hospital. Hemoglobin A1c (HbA1c) was quantified using the high-performance liquid chromatography analyzer Tosoh-G7 (Tosoh Bioscience Inc., Tokyo, Japan). Serum levels of non-esterified fatty acids (NEFA) were measured using the Wako NEFA-HR(2) reagent, which is an enzymatic colorimetric method assay. Fasting insulin concentrations were measured using a chemiluminescent immunoassay (LIAISON, DiaSorin, Saluggia, Italy). Homeostasis model assessment (HOMA-IR) score was used for estimating insulin resistance. Plasma high-sensitivity C-reactive protein (hs-CRP) concentration was measured with an immunonephelometric assay on a Beckman Coulter Image Immunochemistry System (Beckman Coulter, Brea, USA). Low-density lipoprotein (LDL) cholesterol was calculated using the Friedewald’s equation. Glomerular filtration rate (e-GFR) was estimated using the Chronic Kidney Disease Epidemiology Collaboration (CKD-EPI) equation [17]. A pre-existing history of IHD was defined as a documented history of myocardial infarction, angina pectoris or coronary revascularization procedures. The presence of diabetic retinopathy, diagnosed with fundoscopy after pupillary dilation, was also recorded in all patients.

### 2.3. Plasma Ceramides

Blood samples for ceramide measurements were placed into ethylenediamine tetra-acetic acid (EDTA)-containing tubes. Plasma was stored at −80 °C until analysis. An expert laboratory technician (GL), who was blinded to all clinical and biochemical details of participants, performed the measurement of plasma ceramides. Ceramide standards were purchased from Avanti Polar Lipids Inc. (Alabaster, Alabama, USA). Plasma concentrations of Cer(d18:1/16:0), Cer(d18:1/18:0), Cer(d18:1/20:0, Cer(d18:1/22:0), Cer(d18:1/24:0) and Cer(d18:1/24:1) were quantified with liquid–liquid extraction with 2-propanol:ethyl acetate (4:1 *v*/*v*) and gradient reverse phase chromatography on an Agilent Poroshell 120 C18 column (4.6 × 50 mm, 2.7 µm). Cer(d18:1/17:0) was used as an internal standard. The apparatus consisted of an Agilent 1290 UHPLC (ultra-high-performance liquid chromatography) system coupled with an Agilent 6495 Triple Quadrupole liquid chromatography/mass spectrometry (LC/MS) system. Mobile phases consisted of LC/MS grade water (A), acetonitrile with 0.1% formic acid (B) and 10 mM ammonium acetate in 2-propanol (C). [M+H]+→264 MRM (multiple reaction monitoring) transition was selected to quantify each ceramide. Calibration standards (six points) were prepared each day in a surrogate matrix (5% bovine serum albumin) at concentrations ranging from 1.0 to 0.031 µM/L for Cer(d18:1/16:0), Cer(d18:1/18:0) and Cer(d18:1/20:0), and from 10 to 0.31 µM/L for Cer(d18:1/22:0), Cer(d18:1/24:0) and Cer(d18:1/24:1), respectively. Linearity regression coefficients were R2 >0.99 for all ceramides. Inter-assay and intra-assay coefficients of variation for precision and accuracy for all measured ceramides were <15% [2]. No matrix interference or carryover was observed.

### 2.4. Plasma Leucine-Rich α-2 Glycoprotein 1

Blood samples for plasma LRG1 measurement were collected into ethylenediamine tetra-acetic acid (EDTA)-containing evacuated blood tubes and stored at −80 °C until analysis. An expert laboratory technician, who was blinded to all clinical and biochemical details of participants, performed plasma LRG1 measurements. Specifically, plasma LRG1 concentrations were measured using the MILLIPLEX^®^ MAP Human CVD Magnetic Bead kit (Abcam, Cambridge, UK); intra-assay coefficient of variation <5% and inter-assay coefficient of variation <10%, respectively.

### 2.5. Statistical Analysis

Owing to the exploratory design of the study, we did not perform *a priori* sample size calculation. Continuous variables were expressed as means ± SD or medians (interquartile ranges [IQR]), and categorical variables as proportions. The Spearman’s rank correlation analysis was used to test the associations between plasma ceramides, LRG1 and the main clinical and metabolic variables. We used the chi-squared test for categorical variables, the one-way analysis of variance (ANOVA) for normally distributed continuous variables, or the Kruskal–Wallis test for non-normally distributed variables (i.e., duration of diabetes, triglycerides, insulin, HOMA-IR score and plasma ceramides) to examine the differences in the main clinical and biochemical characteristics among postmenopausal women with T2DM, stratified by plasma LRG1 tertiles (1st tertile ranging from 4.5 to 7.5 µg/mL [*n* = 33], 2nd tertile from 7.6 to 9.2 µg/mL [*n* = 33] and 3rd tertile from 9.3 to 15.0 µg/mL [*n* = 33], respectively). The associations between each plasma ceramide (i.e., included as the dependent variable) and LRG1 tertiles (1st tertile vs. 2nd and 3rd tertiles combined) were also tested using both an unadjusted linear regression model and two progressive multivariable regression models. The first multivariable regression model was adjusted for age, BMI and statin use (model 1). The second model was additionally adjusted for systolic blood pressure, HbA1c, plasma total cholesterol level and HOMA-IR score (model 2). We also performed a stepwise linear regression analysis with a forward selection of covariates included in model 2, in which the significance level for addition to the model was set at *p* < 0.05. Covariates included in these regression models were selected as potential confounding factors based on their biological plausibility. To avoid multicollinearity problems in these regression models, we did not also include plasma hs-CRP level as a covariate, because this proinflammatory biomarker was inter-correlated with plasma LRG1 level (as shown in Supplementary Table S1). Although this is an exploratory study, where an adjustment for multiplicity in regression analyses is not mandatory, we performed the Benjamini–Hochberg step-up procedure for controlling the false discovery rate (FDR) (with a q-value of 0.05) in fully adjusted regression models [18]. All statistical tests were 2-sided and a *p*-value of <0.05 (two-tailed) was considered statistically significant. Statistical analyses were performed using STATA software, version 16.1 (STATA, College Station, TX, USA).

## 3. Results

### 3.1. Characteristics of Participants

Table 1 shows the clinical and biochemical characteristics of elderly postmenopausal women with non-insulin-treated T2DM (mean age 72 ± 8 years, mean BMI 29.5 ± 5.0 kg/m^2^, mean HbA1c 6.9 ± 0.7% and median duration of diabetes 10 [IQR: 6–17] years), who were stratified by plasma LRG1 tertiles. Systolic blood pressure and circulating levels of Cer(d18:1/16:0), Cer(d18:1/18:0), Cer(d18:1/20:0) and Cer(d18:1/24:1) increased significantly across tertiles of plasma LRG1 levels. Pulse pressure, a marker of arterial stiffness, also tended to increase across LRG1 tertiles. Plasma Cer(d18:1/22:0) and Cer(d18:1/24:0) levels did not differ significantly among the three patient groups. Similarly, age, BMI, duration of diabetes, smoking history, diastolic blood pressure, HbA1c, HOMA-IR score, plasma lipid profile, renal function parameters, as well as the prevalence of hypertension, IHD or diabetic retinopathy, and the current use of glucose-lowering, anti-hypertensive or lipid-lowering drugs were not significantly different among the three groups of patients.

### 3.2. Univariable Associations of Plasma LRG1 with Plasma Ceramides and Other Clinical and Metabolic Variables

**Figure 1:**
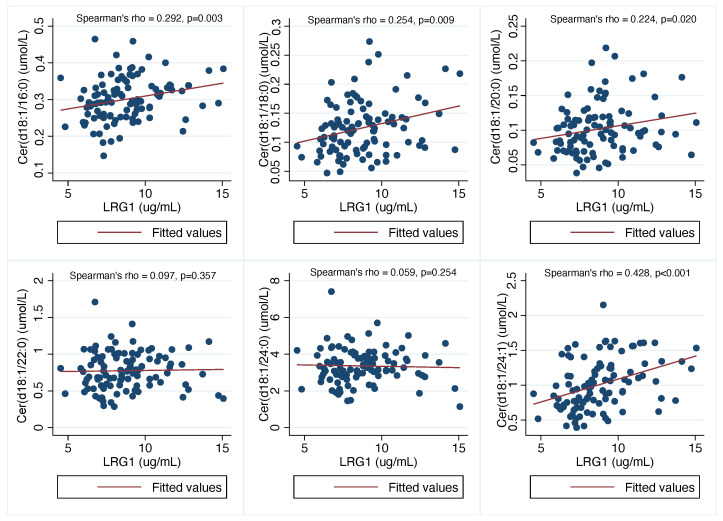
Univariable linear correlations of levels of plasma Cer(d18:1/16:0), Cer(d18:1/18:0), Cer(d18:1/20:0), Cer(d18:1/22:0), Cer(d18:1/24:0) and Cer(d18:1/24:1) with circulating levels of LRG1 in postmenopausal women with non-insulin-treated T2DM (*n* = 99). These correlations were tested using the Spearman’s rank-order correlation analysis.

As shown in Figure 1, there were significant and positive associations between specific plasma ceramide levels [i.e., plasma Cer(d18:1/16:0), Cer(d18:1/18:0), Cer(d18:1/20:0) and Cer(d18:1/24:1)] and plasma LRG1 concentrations in the whole sample of postmenopausal women with T2DM. Conversely, no significant associations were found between plasma Cer(d18:1/22:0) or Cer(d18:1/24:0) and plasma LRG1 concentrations. Supplementary Table S1 shows the Spearman’s rank correlation matrix among plasma ceramides, LRG1 level and other clinical or metabolic variables in the whole patient sample.

### 3.3. Unadjusted and Multiple-Adjusted Associations between Plasma LRG1 Level and Each Plasma Ceramide Measured

The unadjusted and adjusted associations between each measured plasma ceramide and plasma LRG1 concentrations (included as 1st tertile vs. 2nd and 3rd tertiles combined) are shown in Table 2. In unadjusted linear regression models, higher plasma LRG1 levels were significantly associated with higher plasma Cer(d18:1/16:0), Cer(d18:1/18:0), Cer(d18:1/20:0) and Cer(d18:1/24:1) levels. Notably, these associations remained statistically significant after adjusting for age, BMI and statin use (model 1), and even after further adjustment for systolic blood pressure, total cholesterol level, HbA1c and HOMA-IR score (model 2). These results remained essentially unchanged even after adjusting for multiplicity using the Benjamini–Hochberg procedure, with stronger significant associations between higher plasma LRG1 tertiles and increased levels of plasma Cer(d18:1/16:0), Cer(d18:1/18:0) and Cer(d18:1/24:1). The results did not change even when we repeated the aforementioned multivariable linear regression models, including log-transformed plasma LRG1 concentrations as a continuous measure (instead of LRG1 tertiles). Similar findings were also observed when we repeated the aforementioned analyses after excluding patients treated with pioglitazones (*n* = 4) (data not shown). Finally, we also performed a stepwise linear regression analysis with a forward selection of covariates included in model 2 (Supplementary Table S2). Also in such case, higher plasma LRG1 levels were associated with higher plasma Cer(d18:1/16:0), Cer(d18:1/18:0), Cer(d18:1/20:0) and Cer(d18:1/24:1) levels. Conversely, higher total cholesterol levels and HOMA-IR scores were associated with higher plasma Cer(d18:1/22:0) levels, whereas higher values of total cholesterol and HbA1c were associated with higher plasma Cer(d18:1/24:0) levels.

## 4. Discussion

The main findings of our pilot exploratory study, involving elderly postmenopausal women with metabolically well-controlled T2DM, are as follows: (i) compared with those belonging to the 1st tertile of plasma LRG1 levels, postmenopausal women belonging to the 2nd or 3rd LRG1 tertiles had significantly higher levels of previously identified high-risk plasma ceramides, i.e., higher levels of plasma Cer(d18:1/16:0), Cer(d18:1/18:0), Cer(d18:1/20:0) and Cer(d18:1/24:1); and (ii) the observed positive associations between plasma LRG1 levels and these four high-risk plasma ceramides, which have been associated with increased cardiovascular risk, remained statistically significant even after adjusting for age, body mass index, systolic blood pressure, diabetes-related variables, plasma cholesterol level and statin use.

To our knowledge, this is the first cross-sectional study to show a positive and significant association between specific plasma ceramides (which have been associated with increased cardiovascular risk [12,15,19,20]) and circulating levels of LRG1, which is a novel and multifunctional proinflammatory signaling molecule, in a sample of postmenopausal women with metabolically well-controlled T2DM. Specifically, LRG1 is composed of 312 amino acids (66 of which are leucine), containing eight leucine-rich repeats (LRR), four N-linked, one O-linked glycosylation site and two disulphide bonds; this glycoprotein is produced both systemically and at the local tissue level after various inflammatory stimuli, such as infections, injuries and mediators of the acute-phase response (namely CRP, tumor necrosis factor-α or interleukin-6) [3,21,22]. Hepatocytes, neutrophils and endothelial cells are the main cellular sources of LRG1 production [3,22]. Experimental data indicate that LRG1 plays a central role in multiple proinflammatory pathways (mostly modulating the transforming growth factor (TGF)-β non-canonical pathway), as well as in cell survival, protein–protein interaction, cell adhesion and also in some specific metabolic pathways [3]. In this setting, for example, LRG1 may promote hepatic *de novo* lipogenesis, mainly through activation of sterol regulatory element-binding transcription factor-1, and chronic hyperglycemia via inhibiting the insulin receptor substrate (IRS) 1 and 2 expression [23]. In addition, LRG1 is a key player in pathogenic angiogenesis and is associated with circulatory endothelial dysfunction and arterial stiffness in subjects with T2DM [10].

Growing evidence suggests that specific ceramides [especially higher levels of plasma Cer(d18:1/16:0), Cer(d18:1/18:0) and Cer(d18:1/24:1)] are significant predictors for the development of CVD [12] and other chronic inflammation-associated conditions [11], mostly through multiple biological mechanisms, such as induction of endoplasmic reticulum stress, apoptosis, autophagy and increased production of several proinflammatory cytokines [11]. In this regard, ceramides can activate c-Jun N-terminal kinase, nuclear factor-kB and toll-like receptors that trigger multiple proinflammatory pathways, including the TGF-β signaling pathway [11]. Hence, the identification of a significant association between specific plasma ceramides and some proinflammatory biomarkers, such as LRG1, may be useful to further explain how these lipid species might contribute to the development and progression of atherosclerosis and chronic inflammation-associated conditions.

To date, there is little information on the association between specific plasma ceramides and proinflammatory biomarkers in individuals with T2DM and those with established or suspected IHD [2,13,14,15]. Using this same sample of postmenopausal women with T2DM, we have previously reported that increased plasma hs-CRP levels were associated with higher levels of plasma Cer(d18:1/16:0), Cer(d18:1/22:0) and Cer(d18:1/24:1), even after adjustment for known cardiovascular risk factors and other potential confounders [2]. In a small cross-sectional study of 13 obese patients with T2DM, Haus et al. reported significant positive associations of plasma tumor necrosis factor-α with levels of plasma Cer(d18:1/18:0) and Cer(d18:1/18:1), but not with plasma Cer(d18:1/20:0), Cer(d18:1/24:0) and Cer(d18:1/24:1) [13]. In another small study of 33 nondiabetic patients with IHD, de Mello et al. reported a significant positive association between serum interleukin-6 concentrations and total plasma ceramides [14], thus suggesting that ceramides may contribute to triggering systemic low-grade inflammation in IHD. Laaksonen et al. also reported that plasma hs-CRP concentration was positively associated with plasma Cer(d18:1/16:0), Cer(d18:1/18:0) and Cer(d18:1/24:1) levels in univariable analyses, in two cohorts of 1797 patients with established or suspected IHD [15].

Collectively, the findings of our pilot exploratory study provide further evidence for a possible link between previously identified high-risk plasma ceramide molecules and systemic low-grade inflammation in elderly postmenopausal women with metabolically well-controlled T2DM, who are individuals at high risk of developing major adverse CVD events. Preliminary evidence suggests that LRG1 might be more sensitive and specific as a biomarker of chronic inflammation compared to other plasma inflammatory biomarkers, including hs-CRP [5,24]. This might, at least in part, explain some differences in the associations between plasma ceramides and proinflammatory biomarkers that have been reported by some previous studies [2,13,14,15]. However, further mechanistic studies are required to improve our knowledge of the effects of plasma ceramides with different acyl-chain lengths or specific fat compounds on signaling pathways involved in low-grade inflammation, as well as the link between plasma ceramides and LRG1 levels.

The present study has some limitations that should be mentioned. Firstly, the cross-sectional design of the study limits our ability to establish temporal or causal associations between plasma ceramides and LRG1 levels. Secondly, the sample size of the study was small (*n* = 99) and comprised Caucasian elderly postmenopausal women with metabolically well-controlled T2DM. Thus, these results are only indicative and cannot be extrapolated to other ethnic groups of patients, to men with T2DM, or to patients with uncontrolled glycaemia. On the other hand, this latter limitation represents a specific strength of the study, by showing significant associations between plasma ceramides and LRG1 in the absence of major changes in glycaemia. Thirdly, we did not have an adequate control group of non-diabetic postmenopausal women and, therefore, our results should be considered with some degree of caution. Finally, although we adjusted the results for traditional cardiovascular risk factors, diabetes-related variables and statin use, we cannot exclude the possibility that other unmeasured factors might partly explain the observed associations.

Notwithstanding these limitations, the present study has some important strengths, such as the completeness of database, the adjustment for several confounding factors and the exclusion of patients with a documented history of cirrhosis, cancer, kidney failure or other important comorbid conditions. We believe that the inclusion of patients with such serious comorbidities might have confounded the interpretation of data.

## 5. Conclusions

The results of our pilot exploratory study show, for the first time, that plasma LRG1 concentrations were positively associated with circulating levels of previously identified high-risk plasma ceramide species [especially higher plasma Cer(d18:1/16:0), Cer(d18:1/18:0), Cer(d18:1/20:0) and Cer(d18:1/24:1)] in postmenopausal women with metabolically well-controlled T2DM. Notably, these associations remained significant even after adjusting for age, adiposity measures, HbA1c, HOMA-IR score, total cholesterol levels and statin use. We believe that these results may be of clinical relevance, as they further reinforce the existence of a link between previously identified high-risk plasma ceramide molecules (which have been associated with increased cardiovascular risk) and systemic low-grade inflammation. Specifically, our study can shed light on the potential mechanisms by which specific plasma ceramides may promote the development of accelerated atherosclerosis, thereby suggesting that ceramides could become the target of new therapeutic approaches in the foreseeable future. However, given that this is a pilot exploratory study, our findings can be only considered as clues suggestive of the presence of significant associations between plasma LRG1 concentrations and circulating levels of previously identified high-risk plasma ceramide species. Therefore, further larger studies are certainly needed to further corroborate these data in other independent cohorts of individuals with and without T2DM, and for better elucidating the complex biological mechanisms underpinning the association between plasma LRG1 levels and specific plasma ceramides, especially those with long acyl-chain lengths and unsaturated compounds.

## Figures and Tables

**Table 1:** Main clinical and biochemical characteristics of postmenopausal women with non-insulin treated T2DM, stratified by tertiles of plasma leucine-rich α-2 glycoprotein-1 levels.

	Total Sample (*n* = 99)	1st LRG1 Tertile (*n* = 33; from 4.5 to 7.5 µg/mL)	2nd LRG1 Tertile (*n* = 33; from 7.6 to 9.2 µg/mL)	3rd LRG1 Tertile (*n* = 33; from 9.3 to 15.0 µg/mL)	*p*-Values
Age (years)	72 ± 8	72 ± 7	70 ± 9	73 ± 9	0.322
BMI (kg/m^2^)	29.5 ± 5.0	29.1 ± 3.9	29.2 ± 6.1	30.3 ± 4.8	0.550
Diabetes duration (years)	10 (6–17)	11.5 (5–18)	10.5 (6–18)	10 (6–17)	0.844
Current smokers (%)	15.0	14.7	18.2	12.1	0.935
Systolic blood pressure (mmHg)	139 ± 16	134 ± 14	137 ± 18	146 ± 16	0.030
Diastolic blood pressure (mmHg)	76 ± 9	75 ± 9	76 ± 9	77 ± 9	0.532
Pulse pressure (mmHg)	64 ± 16	61 ± 11	62 ± 17	68 ± 16	0.058
Fasting glucose (mg/dL)	128 ± 27	128 ± 31	127 ± 26	131 ± 23	0.805
Hemoglobin A1c (%)	6.9 ± 0.7	7.0 ± 0.8	6.8 ± 0.7	6.8 ± 0.7	0.580
Total cholesterol (mg/dL)	160 ± 32	155 ± 31	160 ± 35	165 ± 32	0.447
LDL cholesterol (mg/dL)	80 ± 29	72 ± 26	80 ± 30	87 ± 30	0.122
HDL cholesterol (mg/dL)	59 ± 13	62 ± 14	58 ± 13	57 ± 13	0.348
Triglycerides (mg/dL)	108 (79–152)	98 (75–145)	124 (79–164)	103 (82–144)	0.717
ALT (IU/L)	14 ± 7	13 ± 5	16 ± 9	12 ± 4	0.068
Creatinine (µmol/L)	67 ± 15	63 ± 12	69 ± 14	70 ± 19	0.108
e-GFR_CKD-EPI_ (mL/min/1.73 m^2^)	79 ± 16	82 ± 12	78 ± 17	76 ± 19	0.246
Insulin (mU/L)	7.5 (4.2–11.9)	6.8 (3.8–9.8)	10.5 (4.4–14.9)	6.9 (4.2–11.4)	0.294
HOMA-IR score	2.3 (1.3–3.7)	2.1 (1.1–2.8)	3.4 (1.4–5.0)	2.3 (1.3–3.6)	0.497
hs-C reactive protein (mg/L)	1.5 (0.7–3.1)	1.0 (0.5–3.8)	1.3 (0.6–2.9)	2.3 (1.0–5.4)	0.053
NEFA (µEq/L)	648 (509–853)	678 (521–841)	672 (501–915)	649 (503–895)	0.991
LRG1 (µg/mL)	8.4 (7.2–9.7)	6.8 (6.3–7.2)	8.4 (8.0–9.2)	10.8 (9.7–12.4)	ND
Hypertension (%)	85.0	85.3	81.8	87.9	0.835
Ischemic heart disease (%)	12.0	11.8	12.1	12.1	0.997
Diabetic retinopathy, any degree (%)	8.0	8.8	9.1	6.1	0.996
Metformin (%)	78.0	85.3	78.8	69.7	0.304
Sulphonylureas (%)	31.0	20.6	33.3	39.4	0.235
DPP-4 inhibitors (%)	26.0	29.4	21.2	27.3	0.778
GLP-1 receptor agonists (%)	12.0	11.8	9.1	15.2	0.750
SGLT-2 inhibitors (%)	5.0	8.8	6.1	0.0	0.239
Pioglitazone (%)	4.0	5.9	0.0	6.1	0.542
Anti-platelet drugs (%)	48.0	47.1	51.5	45.5	0.878
ARBs or ACE-inhibitors (%)	69.0	73.5	57.6	75.8	0.236
Beta-blockers (%)	32.0	26.5	39.4	30.3	0.509
Diuretics (%)	36.0	32.3	30.3	45.4	0.415
Calcium-channel blockers (%)	19.0	26.5	21.2	9.1	0.179
Statins (%)	78.0	79.4	75.8	78.8	0.955
Plasma ceramides					
Cer(d18:1/16:0) (µmol/L)	0.29 (0.26–0.34)	0.28 (0.24–0.31)	0.31 (0.25–0.36)	0.31 (0.28–0.34)	0.013
Cer(d18:1/18:0) (µmol/L)	0.12 (0.09–0.15)	0.10 (0.08–0.13)	0.13 (0.10–0.17)	0.13 (0.10–0.15)	0.008
Cer(d18:1/20:0) (µmol/L)	0.10 (0.07–0.12)	0.08 (0.07–0.11)	0.11 (0.08–0.15)	0.10 (0.08–0.13)	0.027
Cer(d18:1/22:0) (µmol/L)	0.77 (0.58–0.97)	0.69 (0.53–0.90)	0.81 (0.65–1.01)	0.79 (0.58–0.97)	0.279
Cer(d18:1/24:0) (µmol/L)	3.31 (2.82–3.93)	3.15 (2.59–3.77)	3.58 (2.91–3.99)	3.27 (2.84–3.92)	0.531
Cer(d18:1/24:1) (µmol/L)	0.95 (0.72–1.27)	0.76 (0.63–0.99)	0.95 (0.76–1.22)	1.19 (0.91–1.51)	<0.001

Sample size, *n* = 99. Data are expressed as means ± SD, medians and interquartile ranges (in parenthesis) or percentages. Differences among the three patient groups were tested using one-way ANOVA for normally distributed variables, the Kruskal–Wallis test for non-normally distributed variables (i.e., diabetes duration, triglycerides, insulin, HOMA-IR score, C-reactive protein, NEFA and ceramides), or the chi-squared test for categorical variables. Abbreviations: ACE, angiotensin-converting-enzyme; ALT, alanine aminotransferase; ARB, angiotensin II receptor blocker; BMI, body mass index; Cer, ceramide; DPP-4, dipeptidyl peptidase-4; e-GFR, estimated glomerular filtration rate; GLP-1, glucagon-like peptide-1; LRG1, leucine-rich-α2 glycoprotein 1; HOMA-IR, homeostasis model assessment-insulin resistance; NEFA, non-esterified fatty acids; ND; not determined; SGLT-2, sodium/glucose cotransporter-2.

**Table 2:** Unadjusted and adjusted linear associations between each measured plasma ceramide and plasma LRG1 levels (included as 1st tertile vs. 2nd and 3rd tertiles combined) in postmenopausal women with non-insulin-treated T2DM.

Linear Regression Models	Standardized β Coefficient(s)	*p*-Values
**Log Cer(d18:1/16:0)**		
Unadjusted model		
LRG1 (1st tertile vs. 2nd and 3rd tertiles combined)	0.289	**0.004**
Adjusted model 1		
LRG1	0.282	0.004 *
Age (years)	−0.050	0.611
BMI (kg/m^2^)	0.023	0.816
Statin use (yes vs. no)	−0.166	0.098
Adjusted model 2		
LRG1	0.227	0.014 *
Age (years)	−0.021	0.981
BMI (kg/m^2^)	0.029	0.779
HbA1c (%)	−0.068	0.482
Total cholesterol (mg/dl)	0.442	**<0.001**
Log HOMA-IR score	−0.043	0.686
Systolic blood pressure (mmHg)	0.032	0.734
Statin use (yes vs. no)	−0.026	0.786
**Log Cer(d18:1/18:0)**		
Unadjusted model		
LRG1 (1st tertile vs. 2nd and 3rd tertiles combined)	0.307	**0.002**
Adjusted model 1		
LRG1	0.301	0.003 *
Age (years)	−0.104	0.290
BMI (kg/m^2^)	0.089	0.372
Statin use (yes vs. no)	0.035	0.724
Adjusted model 2		
LRG1	0.264	0.008 *
Age (years)	−0.066	0.519
BMI (kg/m^2^)	0.068	0.534
HbA1c (%)	−0.043	0.680
Total cholesterol (mg/dL)	0.283	**0.008**
Log HOMA-IR score	0.503	0.659
Systolic blood pressure (mmHg)	−0.028	0.781
Statin use (yes vs. no)	0.116	0.266
**Log Cer(d18:1/20:0)**		
Unadjusted model		
LRG1 (1st tertile vs. 2nd and 3rd tertiles combined)	0.261	**0.009**
Adjusted model 1		
LRG1	0.256	0.010 *
Age (years)	−0.059	0.554
BMI (kg/m^2^)	0.095	0.349
Statin use (yes vs. no)	0.115	0.257
Adjusted model 2		
LRG1	0.212	**0.034**
Age (years)	−0.022	0.834
BMI (kg/m^2^)	0.062	0.578
HbA1c (%)	−0.049	0.634
Total cholesterol (mg/dL)	0.318	**0.003**
Log HOMA-IR score	0.081	0.483
Systolic blood pressure (mmHg)	−0.018	0.860
Statin use (yes vs. no)	0.203	0.052
**Log Cer(d18:1/22:0)**		
Unadjusted model		
LRG1 (1st tertile vs. 2nd and 3rd tertiles combined)	0.139	0.168
Adjusted model 1		
LRG1 tertiles	0.129	0.195
Age (years)	−0.197	0.051
BMI (kg/m^2^)	0.045	0.663
Statin use (yes vs. no)	−0.085	0.405
Adjusted model 2		
LRG1	0.085	0.327
Age (years)	−0.122	0.176
BMI (kg/m^2^)	−0.081	0.406
HbA1c (%)	0.127	0.169
Total cholesterol (mg/dL)	0.515	**<0.001**
Log HOMA-IR score	0.244	**0.017**
Systolic blood pressure (mmHg)	−0.062	0.493
Statin use (yes vs. no)	0.044	0.630
**Log Cer(d18:1/24:0)**		
Unadjusted model		
LRG1 (1st tertile vs. 2nd and 3rd tertiles combined)	0.055	0.584
Adjusted model 1		
LRG1	−0.053	0.604
Age (years)	−0.123	0.237
BMI (kg/m^2^)	−0.039	0.711
Statin use (yes vs. no)	−0.068	0.512
Adjusted model 2		
LRG1	0.014	0.872
Age (years)	−0.013	0.882
BMI (kg/m^2^)	−0.127	0.179
HbA1c (%)	0.163	0.071
Total cholesterol (mg/dL)	0.638	**<0.001**
Log HOMA-IR score	0.151	0.123
Systolic blood pressure (mmHg)	−0.105	0.230
Statin use (yes vs. no)	0.104	0.240
**Log Cer(d18:1/24:1)**		
Unadjusted model		
LRG1 (1st tertile vs. 2nd and 3rd tertiles combined)	0.343	**<0.001**
Adjusted model 1		
LRG1	0.337	0.001 *
Age (years)	0.114	0.232
BMI (kg/m^2^)	0.096	0.323
Statin use (yes vs. no)	−0.155	0.113
Adjusted model 2		
LRG1	0.287	0.003 *
Age (years)	0.138	0.153
BMI (kg/m^2^)	0.096	0.356
HbA1c (%)	−0.119	0.226
Total cholesterol (mg/dL)	0.313	**0.002**
Log HOMA-IR score	−0.022	0.838
Systolic blood pressure (mmHg)	0.045	0.642
Statin use (yes vs. no)	−0.053	0.585

Sample size, *n* = 99. Data are expressed as standardized beta coefficients as tested by linear regression analysis. Each plasma ceramide (logarithmically transformed before statistical analysis) was the dependent variable in each linear regression model. For the sake of clarity, significant *p*-values are highlighted in bold. * These associations remained statistically significant even after adjustment for multiplicity using the Benjamini–Hochberg step-up procedure (with an FDR of 0.05) [18]. Abbreviations: BMI, body mass index; Cer, ceramides; HOMA-IR, homeostasis model assessment-insulin resistance; Log, logarithmic; LRG1, leucine-rich-α2 glycoprotein 1.

## Data Availability

All data from the study are available in the manuscript (in both Tables and Figures) and supplementary material.

## Supplementary Materials

The following supporting information can be downloaded at: https://www.mdpi.com/article/10.3390/biom12070943/s1, Table S1: Spearman’s correlation matrix among plasma ceramides, plasma LRG1 and other clinical/metabolic parameters. Table S2: Forward stepwise linear regression analyses: independent predictors of different plasma ceramide concentrations in postmenopausal women with T2DM.
