# Supplementary material for: Association between Higher Circulating Leucine-Rich α-2 Glycoprotein 1 Concentrations and Specific Plasma Ceramides in Postmenopausal Women with Type 2 Diabetes
Source: Biomolecules. 2022 Jul 5;12(7):943. doi: 10.3390/biom12070943 (PMC9312999; doi:10.3390/biom12070943)
Supplement: Supplementary file 1 [file biomolecules-12-00943-s001.zip › biomolecules-1779002-supplementary.pdf]

SUPPLEMENTARY MATERIAL

Supplementary Table S1. Spearman's correlation matrix among plasma ceramides, plasma LRG1 and other clinical/metabolic parameters.

	**Cer(d18:1/16:0)**	**Cer(d18:1/18:0)**	**Cer(d18:1/20:0)**	**Cer(d18:1/22:0)**	**Cer(d18:1/24:0)**	**Cer(d18:1/24:1)**	**LRG1**	**Age**	**BMI**	**Cholesterol**	**HOMA-IR**	**HbA1c**	**PAS**	**hsCRP**	**NEFA**
**Cer(d18:1/16:0)**	1.00														
**Cer(d18:1/18:0)**	**0.63****	1.00													
**Cer(d18:1/20:0)**	**0.67****	**0.87****	1.00												
**Cer(d18:1/22:0)**	**0.62****	**0.48****	**0.64****	1.00											
**Cer(d18:1/24:0)**	**0.57****	**0.32****	**0.47****	**0.86****	1.00										
**Cer(d18:1/24:1)**	**0.67****	**0.54****	**0.65****	**0.47****	**0.37****	1.00									
**LRG1**	**0.29****	**0.25****	**0.22***	0.10	0.06	**0.43****	1.00								
**Age**	-0.05	-0.14	-0.07	**-0.22***	-0.15	0.09	0.11	1.00							
**BMI**	0.09	0.11	0.10	0.06	-0.05	0.16	0.13	-0.12	1.00						
**Cholesterol**	**0.45****	**0.30****	**0.28****	**0.45****	**0.52****	**0.34****	0.12	-0.14	0.10	1.00					
**HOMA-IR**	-0.01	0.13	0.18	**0.26****	0.14	0.03	0.05	0.01	**0.37****	0.05	1.00				
**HbA1c**	-0.18	-0.07	-0.09	0.08	0.03	-0.19	-0.14	0.01	0.11	-0.16	**0.30****	1.00			
**PAS**	0.10	0.03	0.03	-0.07	-0.11	0.16	**0.22***	**0.23***	0.16	0.12	-0.03	0.09	1.00		
**hs-CRP**	**0.36****	**0.23***	**0.22***	**0.26****	**0.23***	**0.37****	**0.22***	-0.19	**0.27****	**0.23***	0.18	0.08	0.02	1.00	
**NEFA**	0.05	0.03	0.01	0.07	0.02	0.10	0.04	0.17	0.15	0.12	0.04	0.18	0.16	0.01	1.00

Sample size, *n*=99. Data are expressed as Spearman's rho correlation coefficients. For the sake of clarity, significant p-values are highlighted in bold.
**P*-value <0.05; ***P*-value <0.01

Supplementary Table S2. Forward stepwise linear regression analyses: independent predictors of different plasma ceramide concentrations in post-menopausal women with T2DM.

**Forward stepwise linear regression analysis**	**β coefficients (95% confidence intervals)**	***P*-values**
**Log Cer(d18:1/16:0)**		
Forward-stepwise selection procedure<br>*p = 0.0001 (<0.05) adding total cholesterol*<br>*p = 0.0076 (<0.05) adding LRG1*		
Total cholesterol (mg/dl)	0.003 (0.002-0.004)	<0.001
LRG1 (1st tertile vs. 2nd and 3rd tertiles combined)	0.101 (0.027-0.175)	0.008
**Log Cer(d18:1/18:0)**		
Forward-stepwise selection procedure<br>*p = 0.0022 (<0.05) adding LRG1*<br>*p = 0.0057 (<0.05) adding total cholesterol*		
LRG1 (1st tertile vs. 2nd and 3rd tertiles combined)	0.214 (0.068-0.359)	0.004
Total cholesterol (mg/dl)	0.003 (0.001-0.005)	0.006
**Log Cer(d18:1/20:0)**		
Forward-stepwise selection procedure<br>*p = 0.0036 (<0.05) adding total cholesterol*<br>*p = 0.0193 (<0.05) adding LRG1*		
Total cholesterol (mg/dl)	0.002 (0.001-0.005)	0.007
LRG1 (1st tertile vs. 2nd and 3rd tertiles combined)	0.167 (0.027-0.306)	0.019
**Log Cer(d18:1/22:0)**		
Forward-stepwise selection procedure<br>*p = 0.0001 (<0.05) adding total cholesterol*<br>*p = 0.0023 (<0.05) adding HOMA-IR score*		
Total cholesterol (mg/dl)	0.005 (0.004-0.007)	<0.001
Log HOMA-IR score	0.115 (0.04-0.187)	0.002
**Log Cer(d18:1/24:0)**		
Forward-stepwise selection procedure<br>*p = 0.0001 (<0.05) adding total cholesterol*<br>*p = 0.0147 (<0.05) adding HbA1c*		
Total cholesterol (mg/dl)	0.004 (0.003-0.006)	<0.001
HbA1c (%)	0.084 (0.017-0.151)	0.015
**Log Cer(d18:1/24:1)**		
Forward-stepwise selection procedure<br>*p = 0.0002 (<0.05) adding total cholesterol*<br>*p = 0.0011 (<0.05) adding LRG1*		
Total cholesterol (mg/dl)	0.004 (0.002-0.006)	<0.001
LRG1 (1st tertile vs. 2nd and 3rd tertiles combined)	0.241 (0.099-0.382)	0.001

Sample size, *n*=99. Data are expressed as beta coefficients and 95% confidence intervals (in parenthesis) as tested by forward-stepwise selection linear regression analysis. Each plasma ceramide (logarithmically transformed before statistical analysis) was the dependent variable in each forward-stepwise selection linear regression model. The significance level for addition to the model was p-value <0.05. Covariates included in the forward-stepwise selection linear regression models were: LRG1 (1st tertile vs. 2nd and 3rd tertiles combined), age, BMI, HbA1c, total cholesterol level, HOMA-estimated insulin resistance, systolic blood pressure and statin use.

Abbreviations: Cer, ceramides; HOMA-IR, homeostasis model assessment-insulin resistance; Log, logarithmic; LRG1, leucine-rich-α2 glycoprotein 1.
